# Hospital Notes

**Published:** 1897-03-20

**Authors:** 


### The Institutional Workshop.

### HOSPITAL NOTES.

### HOSPITAL SATURDAY AND ITS SYSTEM OF DISTRIBUTION.

Mr. Reginald B. D. Acland, chairman of the Hospital Saturday Fund, has republished his address delivered at the annual meeting of the Fund on the 27th ultimo. He has often advocated the general adoption by other funds of the plan of distribution devised by his committee, but this address certainly shows the undesirability of any such proposal. In the course of his address Mr. Acland says that the present system of distributing the economy marks by dividing the hospitals into groups and taking averages has worked out so that the awards made for economy to the London Hospital and to the Queen's Jubilee Hospital are respectively £41 10s. 11½d. and £30 4s. 4d. He describes this result as *curious*. We venture to say it is abominable, and that it proves the injustice and unsoundness of the system of distribution which at present prevails in regard to the funds placed at the disposal of the Hospital Saturday Fund Council. Both in the columns of *Truth* and in our own columns it has been proved to the hilt that the Queen's Jubilee Hospital is not a hospital in any proper meaning of that term, and it is past our understanding how an educated man can consent to have his name identified with a system of distribution which not only gives a grant to such an institution as the so-called Queen's Jubilee Hospital, but in practice awards to it a sum for economy very nearly equal to the money granted for economy by the Hospital Saturday Fund to the largest and, on the whole, probably the best administered hospital in the country. Mr. Acland, it is true, desires now to change the present system, but what of the scandalous awards here pointed out?

### THE RE-ENDOWMENT OF GUY'S HOSPITAL.

The interim report on the initiation and progress of the Re-Endowment and Sustentation Funds 1895-96 is one of the most interesting and instructive documents which have ever been published in the interests of a great charity. Well written, packed full of facts, containing a few letters, including one from Mr. W. E. Gladstone, of great literary ability and convincing force, this interim report is a document which should silence for all time those cavillers who are ever harping on the string that the support extended to voluntary hospitals, and especially to the metropolitan hospitals, through the free-will offerings of the people is a decaying quantity and must one day land them on the rates. The report mainly deals with a period of about six months, during three of which earnest and combined efforts were made by the friends of Guy's Hospital under the powerful guidance of H.R.H. the Prince of Wales to improve its financial condition, and to place it upon a sound basis. The Prince of Wales consented to preside at the first festival dinner ever held in the history of Guy's Hospital, and he not unnaturally desired to make that dinner a record so far as the contributions received were concerned. The report shows that, as a result of these efforts, a capital sum of £282,000 was received and promised. Of this vast sum, £162,000, in round numbers, represents donations, £10,000 represents contributions payable in varying instalments, and £110,000 the capitalised value, at a moderate estimate, of the £3,300 promised in annual subscriptions. And here we would remark on the one blot which the report displays, namely, the absence of any balance-sheet or audited statement showing the cost of raising this money, and of any paragraph pointing out that, of the capital sum of £500,000 asked for, £282,000, or nearly three-fifths of the whole, has been received. The figures as they stand must be intensely gratifying to the Prince of Wales and to everybody who is interested in Guy's Hospital, as well as to all who take any part in the endeavour which has been steadily gaining force in London during the last twenty years, to place the voluntary principle of hospital support upon so sound a financial basis as to make it secure from the attacks of those thoughtless and ignorant persons who advocate rate support for the great voluntary hospitals of our country.

### FROM ENDOWMENT TO VOLUNTARY SUPPORT.

This interim report of Guy's Hospital brings once more to mind the true principle upon which the maintenance and efficiency of our voluntary hospitals must ever depend. Experience proves that a huge endowment makes rather for inefficiency than in favour of progress and the highest administration. Where an institution has so much invested property, and its managers feel themselves to be above public criticism, there in process of time will relative inefficiency, red tape, and other impediments to progress make their appearance, to the injury and peril of the very existence of the work which the original founders had so much at heart. Guy's Hospital, thanks largely to the public-spirited efforts of the Prince of Wales, has now taken its place amongst the great voluntary hospitals of London, and it is not ashamed to recognize—and it is of the first importance to Guy's Hospital that its governors have been brought to this view—that they must look henceforward year by year to the public for a definite proportion of the requisite income to maintain the whole institution in efficiency and to keep all the available beds fully at work. At the present time Guy's Hospital has 150 beds closed, and in order to enable the beds now open to be kept going, and the closed beds to be made available for the succour and treatment of the sick poor, an additional income from voluntary sources of about £15,000 will be required each year. We would counsel the governors to drop their present plan of advertising for contributions to an endowment fund of £500,000, to which they state they have received £162,000, instead of the £282,000 actually provided. The public very rightly are opposed to further endowment for Guy's or any other institution of the kind. If, however, this misleading and inaccurate statement is dropped out of the appeals altogether, and the governors substitute for it a plain statement to the effect that if £15,000 a year more is forthcoming in voluntary contributions from the public, they will be able to open 150

more beds, and to keep the whole of Guy's Hospital going with the maximum efficiency, we make no doubt that this £15,000 will be forthcoming within the next five years.

### LONDON SOUTH OF THE THAMES.

From a statement issued by Mr. Wainwright, the treasurer of St. Thomas's Hospital, we find that in 1894 London south of the Thames, the district which sends more patients to Guy's than any other, with its population of upwards of one million and a-half, at present contributes in voluntary contributions and legacies to the metropolitan hospitals only £43,229. London north of the Thames, with a population of 2,687,000, contributes in voluntary contributions and legacies to the metropolitan hospitals no less than £386,282. That is to say, whereas every thousand persons resident in London north of the Thames give every year £143 to the London hospitals, the residents south of the Thames contribute considerably less than £30 a year per thousand to the same institutions. This astounding state of affairs is due no doubt mainly to the circumstance that the people resident in South London have for many decades relied mainly upon the endowed hospitals, Guy's and St. Thomas's, for the hospital relief of their sick poor. Both Guy's and St. Thomas's Hospitals are at the present time in need of additional funds, and if they will devote their attention mainly to the people who reside in London south of the Thames they should find little difficulty in quickening the interests of the sluggards who have been so long left without appeals and opportunities to contribute to the hospitals, to their loss and to the infinite increase of avoidable suffering throughout South London. If the people who reside south of the Thames were to bring their contributions to hospitals up to the same average sum per thousand as that represented by the contributions of the residents north of the Thames, not only would St. Thomas's and Guy's Hospitals be placed in abundance of funds, but the people of South London might have one or more outpost or independent general hospitals in their midst, to which the poor might resort, as the residents in North London now resort to the Great Northern Central Hospital. The burden imposed upon other hospitals situated outside this district would be thus immeasurably lessened, to the credit of the population, who have been so long in default, and to the benefit of the numerous class of poor people who have been largely neglected and overlooked by the wealthier residents south of the Thames during the past century. It has been urged in defence of the position of affairs here brought to light that the population south of the Thames is infinitely poorer than that resident in other parts of London. We have an intimate knowledge of the circumstances of the people in South London, and we are confident that they are perfectly well able to contribute at least £150 per thousand per annum to the support of the metropolitan hospitals, and that such a contribution would not only not be felt by the givers, but would provide more than all the money which is necessary to place Guy's and St. Thomas's Hospitals, and any other institution which it may be thought desirable to establish in South London for the relief of the sick upon a sound financial basis, and to leave a substantial surplus to meet any emergencies or further requirements which circumstances and experience may prove to be necessary.

### THE ROYAL SURREY COUNTY HOSPITAL AND HOME VISITING.

A question in hospital management has been raised at Guildford which should receive the careful consideration of all concerned. The question is as to the propriety of resuming the home visiting, which in consequence of a resolution passed some time ago by the governing body has lately been abolished. It is to be noted that the matter is somewhat complicated by the fact that years ago the hospital took over the work of a dispensary which then existed in the town, receiving from it certain moneys on condition of carrying on its work. Into this, which is a purely local affair, we cannot enter. We would limit ourselves, therefore, to the main question as to the propriety and advantage of maintaining a home visiting dispensary in a town which already has a hospital with a considerable out-patient department, in addition to the Poor Law arrangements of workhouse and district medical officers. The whole thing hinges on the possibility of working the "letter system" without abuses creeping in, and all experience teaches that to work this system without abuses is impossible.

We are quite ready to admit that there are many poor people in every town who, while not paupers, are unable to pay the ordinary fees of medical men, and we quite see that to the charitably-disposed it appears the simplest thing in the world to send the house surgeon of the hospital to visit them. But then we go further, and say that the class of people who are unable to pay ordinary fees, and yet are not paupers, is much larger than could be dealt with by any such scheme, and contains a crowd of honest, working, wage-earning people, who, for all their work, and for all their wages are quite unable to pay a doctor in the ordinary way. For medical attendance on these people no provision whatever is made in many towns, whith the result that when illness comes upon them they are forced either to throw themselves upon charity, and sap their own independence by habituating themselves to the reception of alms, or must sponge upon the medical profession, running up debts that both they and their doctors know well will never be paid, but still will hang like millstones round their necks; or they must become paupers. Yet the great majority of these people could pay for medical attendance easily enough if the mode of payment were so arranged as to suit the circumstances of a weekly-wage-earning population. Far better, then, establish a provident dispensary than a system of free home visiting from the hospital. Those who could not afford the small weekly payment required by a provident dispensary, amounting to about a glass of beer once a fortnight, may really be looked upon as paupers, while the chronics, the old cases of rheumatism and bronchitis, which would hardly be accepted by a self-supporting provident dispensary, might well be dealt with by that charity which is always to be found in every English community. The poor, and even the non-pauper poor, we shall always have with us, and for the relief of the latter we believe that charity will always step in. What we think is a mistake is the establishment of a free system of home visiting, which tends to demoralise a whole town, for the sake of providing home attendance on a few deserving people, who, if a provident dispensary existed, could quite easily be provided for, not by the establishment of a system of "free tickets" alongside of the "pay tickets," but by those who are charitably disposed paying the dispensary subscription for such as seemed worthy of help.

This is a matter which should be at once taken up by the medical men of Guildford. Both charity and self-interest should prompt them to action in the matter. At first sight it may seem to some that the establishment of home visiting from the hospital may relieve medical men in the town of a certain amount of unremunerative work which otherwise tends to be thrown upon them; but, in fact, it has never been found possible under the ticket system to restrict charity to proper objects; and there can be no possible doubt that, with human nature as it is, such a system of free home visiting is sure to be taken advantage of by large numbers of working-people who could well afford to support a provident dispensary. The medical profession unfortunately is at present very jealous of the provident system. The evils that it has wrought in many towns are so manifest that many medical men entirely decline to have anything to do with it. We cannot avoid saying, however, that these evils are to a large extent the medical men's own fault, and are the direct result of their leaving to others the organisation of institutions which they ought in every town to arrange and manage for themselves. If the medical men of Guildford will combine to run a provident dispensary — sinking no money in bricks and mortar, but dispensing from their own surgeries as they do for their private patients—and will run it at the smallest possible subscription, spending all their energies in keeping out unsuitable cases rather than in making the affair especially remunerative, they will bring into their own pockets a small but not absolutely contemptible sum; they will save to themselves a somewhat larger sum by preventing all excuse for the establishment of a system of free visiting from the hospital; they will check the demoralising tendency which is always associated with the acceptance of eleemosynary aid by those who are not in want; and they will make it easy for the charitable to provide, at a very modest rate, for medical attendance on such of their protégés as are otherwise proper persons for dispensary treatment. No doubt the establishment of such an institution involves a little trouble; nevertheless, it is the proper solution of the difficulty; and if the medical profession want it to be a help and comfort to them in their work instead of being a constant thorn and irritation, they must take the trouble and must establish the dispensary themselves, and run it on proper professional lines.

